# Are the resources adoptive for conducting team-based diabetes management clinics? An explorative study at primary health care centers in Muscat, Oman

**DOI:** 10.1017/S1463423618000282

**Published:** 2018-05-08

**Authors:** Kamila Al-Alawi, Helene Johansson, Ahmed Al Mandhari, Margareta Norberg

**Affiliations:** 1 Department of Public Health and Clinical Medicine, Unit of Epidemiology and Global Health, Umea University, Umea, Sweden; 2 Director of Training and Studies, Department of Training and Studies, Royal Hospital, Ministry of Health, Muscat, Oman; 3 Senior Research Assistant, Department of Public Health and Clinical Medicine, Unit of Epidemiology and Global Health, Umea University, Umea, Sweden; 4 Senior Consultant, Department of Family Medicine and Public Health, Sultan Qaboos University Hospital, Muscat, Oman; 5 Associate Professor, Department of Public Health and Clinical Medicine, Unit of Epidemiology and Global Health, Umea University, Umea, Sweden

**Keywords:** diabetes care, Oman, perception, primary health care, team-based management

## Abstract

**Aim:**

The aim of this study is to explore the perceptions among primary health center staff concerning competencies, values, skills and resources related to team-based diabetes management and to describe the availability of needed resources for team-based approaches.

**Background:**

The diabetes epidemic challenges services available at primary health care centers in the Middle East. Therefore, there is a demand for evaluation of the available resources and team-based diabetes management in relation to the National Diabetes Management Guidelines.

**Method:**

A cross-sectional study was conducted with 26 public primary health care centers in Muscat, the capital of Oman. Data were collected from manual and electronic resources as well as a questionnaire that was distributed to the physician-in-charge and diabetes management team members.

**Findings:**

The study revealed significant differences between professional groups regarding how they perceived their own competencies, values and skills as well as available resources related to team-based diabetes management. The perceived competencies were high among all professions. The perceived team-related values and skills were also generally high but with overall lower recordings among the nurses. This pattern, along with the fact that very few nurses have specialized qualifications, is a barrier to providing team-based diabetes management. Participants indicated that there were sufficient laboratory resources; however, reported that pharmacological, technical and human resources were lacking. Further work should be done at public primary diabetes management clinics in order to fully implement team-based diabetes management.

## Background

Globally, diabetes is a fast-growing epidemic (Guariguata *et al*., 2014; Unnikrishnan *et al*., 2017). Arabic speaking countries, specifically the Gulf Council Countries (GCCs), are considered leaders in this epidemic (Alhyas *et al*., 2011). The International Diabetes Federation (IDF) has estimated that around 32.8 million (9.1%) individuals in the Middle East and North Africa have type 2 diabetes (Whiting *et al*., 2011; Al-Yaarubi *et al*., 2015). Oman is in a very frontal place (Badran and Laher, 2012) with a recorded prevalence of 20% in one of its regions in 2010 (Al-Lawati *et al*., 2015). Diabetes is considered the second most common chronic disease in Oman after cardiovascular diseases (Al-Moosa *et al*., 2006; Al-Lawati *et al*., 2008; 2015). According to the World Health Organization (WHO), Oman will witness an almost threefold increase in patients with diabetes over the coming years, rising from 75 000 to 217 000 by year 2025 (Al-Shookri *et al*., 2011). With this fast expansion, actions related to high quality, team-based approaches for diabetes management at primary health care centers must be taken rapidly.

Worldwide, research has shown that interdisciplinary teams provide better clinical outcomes and higher patient satisfaction (Grumbach and Bodenheimer, 2004). Furthermore, patients with diabetes have growing demands and need effective primary care teams without losing the benefits of comprehensiveness, competence and continuity (Wagner, 2000). In the GCCs, the awareness of non-communicable diseases, such as diabetes, is growing. Though providing team-based management for these diseases is crucially important (Klautzer *et al*., 2014), challenges facing the system have been reported. For example in the Kingdom of Saudi Arabia, poor dissemination of guidelines in primary care settings and inadequate implementation of evidence-based medicine has been reported; this is in addition to challenges related to organization at primary health care centers (Hanan and Roland, 2005). Similarly, in the United Arab Emirates challenges related to teamwork, the developing role of nurses in diabetes care, and improving information systems at the primary health care level are recognized (Khattab *et al*., 2007).

Health care teams in primary care require special attention and should focus on who is on the team and how team members work together (Grumbach and Bodenheimer, 2004). A simple definition of team is a group of individuals who collaboratively work on specific tasks to accomplish a shared goal (Grumbach and Bodenheimer, 2004). The chronic care model, which is the ultimate model in diabetes management for primary health care, focuses on nurse-led clinics. This model is widely used and applied around the world (Wagner *et al*., 2001; Bodenheimer *et al*., 2005). Furthermore, strong leadership and appropriate patient access to health care are essential requirements for team-based approaches (McGill *et al*., 2017). Additional elements for functioning teams are key elements of team building, including defined systems, training and appropriate feedback processes. The diabetes teamwork in primary care does not only require an accurate assessment of glycemic control (McGill and Felton, 2007) and increased understanding of diabetes care, but also the ability to work collaboratively to create an environment that promotes trust and supportive relationships (McGill *et al*., 2017).

According to WHO and IDF, diabetes management core teams should consist of the patient, a physician, and a diabetes educator. The diabetes educator may be a specialist diabetes nurse, dietician, or other health care professional. This core team structure has been shown to be essential to the provision of quality diabetes care and education. Depending on the resources of the country, additional team members may be added to the core team. Therefore, team-based diabetes management approaches and task distribution vary across locations (Aschner *et al*., 2007a). In Oman, the team consists of a physician, a nurse, a dietician, and a health educator.

The interaction between primary health care providers and patients with type two diabetes in Muscat, Oman has been explored through several studies (Abdulhadi *et al*., 2006; Abdulhadi *et al*., 2007; Abdulhadi *et al*., 2013) and suggested that there are lack of teamwork approach, dissatisfaction in diabetes management service and compliance with guidelines need further to be explored.

Based on the current research evidence related to prevention and treatment of diabetes, as described in the IDF Guidelines (IDF, 2012; 2015), American Association of Clinical Endocrinology and American College of Endocrinology Diabetes Management Guidelines (Handelsman *et al*., 2015), the Ministry of Health (MOH) in Oman developed the National Diabetes Mellitus Management Guidelines in 1996.The guidelines were updated in 2003 and 2012; the 3rd version was reprinted in 2015. These guidelines present the management of diabetes as a complex process, indicating that the approach requires a free exchange of information between health care providers and patients in supportive and therapeutic environments. A classification of diabetes mellitus with its diagnostic criteria, acute and chronic complications, and treatment is described. This includes measurements and examinations, laboratory support, pharmacological treatment, and patient education. The guidelines recommend an interdisciplinary team-based approach in order to provide high quality care for diabetes management at health centers. This recommendation is supplemented with patient–provider interaction and communication skills. However; the most recent guidelines mainly focus on the physician’s role with very little attention on the other members of the team, their daily roles as decision makers, their achievements or their agreements upon team strategies.

Despite the availability of guidelines in Oman, there are many challenges related to diabetes clinics at primary health care (Alyaarubi, 2011). Although the diabetes management guidelines for primary care are available, it is not clear if the guidelines are applied in primary care practice (Campbell *et al*., 2000).

The aim of this study is to explore attitudes and perceptions among primary health center staff about team-based diabetes management with attention to self-perceived competencies, values and skills, as well as perceived support for team-based diabetes management and to describe the availability of needed resources for the team-based approach. The results of the study will be used by the Oman MOH to establish a long-term strategic plan to tackle non-communicable diseases including diabetes mellitus.

## Methods

### Design of the study

This study is part of a larger project exploring the feasibility of interdisciplinary teams in the management of diabetes at the primary health care level in Muscat, the capital of Oman. This cross-sectional study was conducted between March and April 2015.

### Setting

Oman is a GCC with a population of 4 414 051people; immigrants make up more than 40% (Ministry of Health, 2016). The median age is 25 and the majority of the population lives in Muscat with a mixed ethnicity group of Arab, Baluchi, South Asian (Pakistani, Sri Lankan, Bangladeshi) and African. The official language is Arabic beside English, Baluchi, Urdu, and Indian idioms (Ministry of Health, 2016).

The health care sector in Oman is divided into primary, secondary, and tertiary health care and is delivered under public and private territories. The private clinics provide basic primary health care that involves a single physician, rather than multidisciplinary, approach, does not include the patient as a member of the team, and does not encourage the use of additional support networks. It was found that only 22% of them are specialized to provide diabetes management care (MOH-Oman); 70% of patients with diabetes obtain health care from public health centers, 19% from traditional healers and 11% from the private sector (Al-Mandhari *et al*., 2013). The private sector is not within the scope of this study.

Muscat has a total of 27 public primary health care centers, 26 of which provide diabetes screenings, diagnoses, management and follow-up by a four-member team as recommended by the national guidelines. These teams include a physician, diabetes practice nurse, dietitian, and health educator. Complicated diabetes cases are referred to secondary and tertiary health care institutes.

### The instrument

#### Pilot phase

A questionnaire was designed in January 2015 to mirror the requirements for the primary care level of the National Diabetes Mellitus Management Guidelines. It was piloted in eight centers located in the largest province of Muscat. In the pilot phase, the questionnaire was distributed in person to the physician in charge and the members of the diabetic team at each center. Participants provided written comments and suggestions as free text on the questionnaire. The comments were used by the research team to adjust the questionnaire before final distribution.

#### Variables of the instrument

The first section of the questionnaire (Appendix 1, Section A) included questions about the number of patients. Data were collected from available manuals and electronic registries at the health centers by the first author and four field workers.

The physician-in-charge completed the second section of the survey (Appendix 1, Section B and C), which included questions regarding resources (staff and clinics) for diabetes care. It also included availability of resources for screening, diagnosing and monitoring patients, as well as pharmacological treatments and patient education.

The third section (Appendix 1, Sections D, F, H, and J) was a survey and answered individually by the members of the diabetic team: physicians, nurses, dieticians, and health educators. The questions covered gender, age, nationality, qualifications, professional titles, and training in diabetes management. Age was stratified by the median into two groups, age ⩽35 years and >35 years.

This section also contained questions on self-perceived competencies for diabetes management (three items), self-perceived values towards team-based diabetes management (10 items), self-perceived team-related skills (nine items) and self-perceived support and resources for diabetes care (nine items) (Appendix 1, Sections E, G, I, and K). These questions were answered using a Likert scale where completely agree=3, agree=2, disagree=1, and completely disagree=0. In addition, the option ‘Do not know/Not applicable’ was also available. Overall scores for self-perceived competencies, values, skills and resources were calculated by combining ‘completely agree’ and ‘agree’ into ‘agree’ and ‘completely disagree’ and ‘disagree’ into ‘disagree.’ Findings are reported by professional group affiliation and the frequencies and percentages of ‘agree’ and ‘disagree’ for each of the 31 items of the survey (Appendix 2).

### Data collection phase

The distribution and collection of the questionnaire was conducted by the first author and the field workers. The third author assisted with formalities related to distributing the questionnaire; all authors contributed to its development. The first author and the field workers conducted one meeting before questionnaire distribution and one meeting after the data collection. The meetings aimed to train the field workers on the questionnaire, summarize the visits and decide on additional complimentary visits (if necessary). The primary centers were randomly allocated to the first author and field workers who visited them to distribute the questionnaire.

All 26 public primary centers that provide diabetes management service in Muscat under the MOH were approached. To ensure minimum disruption by the study, the questionnaire was given to the physician in charge to answer sections B and C. The physician in charge then distributed the questionnaire to each of the available diabetic team members to answer sections D−K when time permitted, either during or outside of working hours. The questionnaires were only distributed to the four professions that are mentioned in the current national diabetes management guidelines. One week after questionnaires were distributed, the first author and field workers collected the questionnaires (in sealed envelopes) and details on the number of questionnaires distributed, from each center. No losses were recorded and anonymity was preserved.

### Statistical analysis

All statistical analyses were carried out using SPSS (version 22.0 for windows). For categorical variables, frequencies and percentages were reported. For continuous variables, mean and median values were reported with minimum and maximum values. Because the variables were not normally distributed, comparisons between different professional groups’ characteristics (perceived competencies, values, skills, and available resources) were conducted using non-parametric tests, namely the Mann–Whitney *U* and Kruskal–Wallis *H* tests of association. The Mann–Whitney *U* test was used to compare differences between two groups (age, sex, and nationality) and The Kruskal–Wallis *H* test allowed the comparison of more than two groups (staff profession). In both tests, the mean rank values (the average values of the median values) were calculated to estimate the association. A non-parametric Dunn’s *post-hoc* multiple comparison test was performed after a statistically significant Kruskal–Wallis test. A two-tailed level of significance was set at 0.05.

### Ethical approval

The project was approved by the Research and Ethical Review and Approval Committee of the MOH in Oman and was conducted in accordance with the Helsinki Declaration, version (2007–2008).

## Findings

### Section 1

The diabetes management clinics were conducted four to five days per week in mornings and afternoons. A mean of 300 diabetic patients were seen per month in each center. Every patient came to a visit every three months. A complete description of the clinics and the service provision of team-based approach are not within the scope of this study.

A total of 300 primary care physicians (81% females), 411 nurses (99% females), 25 dietitians (100% females) and 20 health educators (100% females) were registered in the selected centers during data collection.

A total of 115 (38%) of the physicians were involved in providing diabetic care. Corresponding numbers for nurses, dieticians, and health educators were 86 (21%), 23 (92%), and 20 (100%), respectively. In addition to the main team members, others including pharmacists, assistant pharmacists, a psychologist, a dentist, and a medical orderly were involved in teams in some centers, but were not included in the survey.

### Section 2

In regard to the measurements and treatment modalities, all 26 health centers measured weight, height, and blood pressure and examined the heart and diabetic foot. Only one center performed funduscopy of the diabetic eye; the others referred the patients to secondary and tertiary institutes. In half of the centers, serum creatinine, and glycosylated hemoglobin (HbA1c) tests were performed while the remaining centers sent the tests to secondary and tertiary institutes. All centers performed random, fasting, and oral glucose tolerance tests as well as blood count. Urine glucose dipsticks and urine albumin tests were performed at all centers andurine albumin creatinine ratios were collected at half of the centers. The health centers that did not have the laboratory support for the recommended tests sent the tests to secondary or tertiary institutes. All 26 centers had oral hypoglycemic, dyslipidemic and hypertensive drugs and provided patient education. A total of 25 centers provided insulin and performed patient and glucose monitoring education. In all, 14 centers provided cardiovascular disease drugs.

### Section 3

Of the 244 staff members providing diabetic care, 212 were available to answer the questionnaire (87%). Respondents included 79% (*n*=91) of physicians, 93% (*n*=80) of nurses, 96% (*n*=22) of dieticians and 95% (*n*=19) of health educators. The 32 staff who were not included in the survey were on official leave. Thus, the response rate among all individuals that were available was (100%).

Among physicians, 73% were females. All nurses, dietitians and health educators were females. The main nationality of physicians was Omani (*n*=51; 90% females); the others were recruited from different Asian and African countries. The other staff included mainly Omanis with only 11 Indian nurses. Among the staff, 79 (37%) had specialized qualifications. Among physicians 56% were specialized; corresponding percentages among nurses, dietitians and health educators were 6%, 65%, and 43%, respectively.

The complete results from section 3 of the survey are given in Appendix 2: Tables A, B, C, and D. Missing data on single items were at most 3% in all professional groups and missing answers were excluded from the analyses.

#### Staff self-perceived competencies

All staff reported high self-perceived competencies on the three items. A maximum score of 100% was reported from health educators regarding having up-to-date skills to conduct diabetes management clinics. A minimum score of 85% was reported from nurses regarding considering oneself a resource person for diabetic patients (Appendix 2: Table A).

#### Staff self-perceived values

Staff believed they were responsible for providing up-to-date knowledge and skills. They also considered patients’ self-management an important part of diabetes management. The minimum scores were recorded among health educators and nurses in regards to their perception of knowledge, skills, and patient self-management, respectively. With the exception of 74% of health educators, the majority of all professions 97% agreed to respect diabetic patients’ beliefs in regards to the disease. Approximately 91% of the staff perceived capacity building, providing the recommended diabetes management and having a good effect on patients’ decision very important with lower recordings among nurses, dietitians, and health educators. Around 75% of physicians, nurses and health educators indicated that the culture of the primary centers helps in providing the recommended diabetes management. By contrast, among dietitians, only 55% agreed on this. Finally, the professional groups agreed to the concept that nationality can effect patients’ decisions (range: 50% of physicians to 91% of dieticians) (Appendix 2: Table B).

#### Staff self-perceived team-related skills:

Across all professional groups, 93% agreed that they considered themselves part of the team with the lowest scores among nurses 88%; 73% of physicians, 64% of nurses, 41% of dieticians, and 61% health educators surveyed considered themselves leaders in the team. The majority of the professionals in each group agreed to the idea of accepting inter-professional relationships in the diabetes team and encouraging teamwork related to diabetes management. Furthermore, the majority of the professionals in each group agreed that they have the ability to reflect on patients’ decisions related to diabetes management and deliver care as part of a team. All professional groups also reported that they value teamwork related to diabetes management and encourage collaboration, though nurses reported lower scores (Appendix 2: Table C).

#### Staff self-perceived support/resources

All professional groups (71%) (range: 41% of dieticians to 84% of physicians) agreed that they have easy access to the internet in order to stay up-to-date on diabetes knowledge. Among all professional groups, 38% agreed (range: 33% of dieticians to 43% of physicians) that they are supported financially in order to update their diabetes knowledge; 91% of participants (range: 86% of dieticians to 92% of physicians) consider their manager and colleagues supportive of continued education in diabetes. In the four professional groups, 76% (range: 45% of dieticians to 83% of physicians) considered the infrastructure and work environment helpful in providing the recommended diabetes management. The physicians (67%, 63%) and nurses (68%, 53%) evaluated human and technical resources respectively lower than the other professional groups, dieticians (77% , 68%) and health educators (79%, 68%), respectively (Appendix 2: Table D).

Sixty-seven percent of Omani staff and 90% of non-Omani agreed that the culture in the primary care centers helps in providing the recommended diabetes management. Furthermore, 71% of Omani staff and 41% of non-Omani staff answered that their nationality could affect patients’ decision related to diabetes management.

Results from comparisons between professional groups with regard to self-perceived competences, values, team-related skills, and support are shown in Table 1.Those who were >35 years old, males or non-Omani believed they had more team-related skills and received more support for team-based diabetes care compared with what younger, female or Omani staff reported. Comparison of the four professional groups showed significant differences in terms of self-perceived competencies, values and team-related skills; physicians scored highest in all three domains.

**Table 1:** Results from a survey among primary health professionals (*n*=212), including physicians, nurses, dieticians and health educators, from 26 primary health centers in Muscat, Oman regarding self-perceived team-based competences, values, team-related skills, and support in relation to age, sex, nationality and type of profession

	Perceptions			
Characteristics	Competencies^a^	Values^b^	Skills^c^	Support^d^
Age	Mean Rank			
⩽35 years	97.0	96.3	87.5	92.7
>35 years	109.2	103.8	111.5	110.3
*P* value^e^	0.124	0.355	0.003	0.031
Sex				
Male	121.2	121.8	127.7	124.6
Female	102.8	99.2	97.8	100.6
*P* value^e^	0.137	0.071	0.016	0.058
Nationality				
Omani	102.4	99.0	93.2	95.3
Non-Omani	113.8	112.0	127.4	129.9
*P* value^e^	0.232	0.182	<0.001	<0.001
Profession				
Physician	122.3	117.3	118.6	114.0
Nurse	93.8	93.3	86.7	97.7
Dietitian	80.0	94.4	83.9	83.5
Health Educator	98.1	77.6	99.0	100.6
*P* value^f^	**0**.**002**	**0**.**011**	**0**.**002**	0.115

Significant results are shown in bold.

a Competences (knowledge, skills, and being a resource person).

b Values (responsibility, patients’ education, patients’ self management, communication, patients’ beliefs, building capacity, commitment, culture, patients’ decision, nationality).

c Team-related skills (team-based diabetes management, part of the team, leadership, inter-professional relationship, reflection, teamwork encouragement, teamwork delivery, teamwork value, collaboration).

d Support (time, internet, financial support, manager support, colleagues support, center infrastructure, work environment, human and technical support).

e Mann–Whitney *U* test.

f Kruskal–Wallis *H* test.

Further analyses with Dunn’s *post-hoc* multiple pairwise comparison test showed that physicians considered themselves to have better competencies than nurses and dieticians (*P*=0.013 and 0.009, respectively). Physicians also scored higher on team-related skills and values compared with health educators (*P*=0.045). In terms of team-related skills, the difference between physicians and nurses was statistically significant (*P*=0.003) and showed that physicians perceived themselves to have better skills than nurses. No other pairwise comparisons were statistically significance.

In 24 primary health care centers with 201 staff involved in diabetes management, the physician-in-charge declared that the staff were aware of the updated National Guidelines for Diabetes Management. At the same time, 23% of staff in those centers indicated that they were not aware of the Guidelines. Despite this, 92% of staff in these centers agreed that they have up-to-date knowledge to conduct diabetes management clinic.

In two primary centers with a total of 11 staff involved in diabetes management, the physician in charge declared that the staff were not aware of National Guidelines for Diabetes Management. In contrast, five of the 11 staff that were involved in the diabetes management team said that they were aware of the Guidelines and 10 out of 11 staff declared that they had up-to-date knowledge to conduct diabetes management clinics.

## Discussion

The findings in this study revealed important areas for improvement in order to fully provide team-based care in diabetes management clinics in primary health care. The findings showed small but significant differences between the four professional groups regarding how they perceived their own competencies, values and skills as well as resources related to team-based diabetes management. These differences could be the bases for improvement.

In terms of self-perceived competencies, the four-member teams perceived their diabetic knowledge and skills very high; all team members considered themselves resource persons. Overall physicians ranked themselves higher than nurses, who had concerns with key team-related factors for diabetes management, including: capacity-building, considering themselves part of the team, having a chance to reflect on patient’s decision, delivering and valuing teamwork and encouraging collaboration. This could be due to a lack of specialized knowledge in diabetes management, as only 6% of nurses were specialized. The lack of specialized nurses and related outcomes are in agreement with previous studies conducted in Muscat (Abdulhadi *et al*., 2006; Abdulhadi *et al*., 2013). These results suggest the necessity of training and education for providers to update their skills. Concerns among nurses could also be due to a shortage of nurses assigned to diabetes management and thus the time they can spend on diabetes management or the organization of their work. Therefore, increase in availability of nurses to diabetes management clinics, or to assign some nurses to diabetes and other to other assignments might be a suitable solution.

While this study was not designed to clarify the different professionals’ roles and tasks in the diabetes teams, it is important to note that considerable differences in qualifications among team members may lead to disentitlements among team members. This can then lead to unequal approaches to patients at diabetes management clinics and may affect which team members the patients will listen to. This could negatively affect the nurses who were the least specialized members of the team.

The different approaches of team members is in line with the fact that the current guidelines provide little, if any, information regarding competences and skills required by team members other than physicians. The guidelines also provide little guidance about which tasks could be shifted from physicians to nurses, dietitians or health educators in team-based settings. This is in contrast with the literature, which indicates that redefining the roles of health care delivery team members and empowering patient self-management are fundamental to successful diabetes team management (ADA, 2017).

In addition, both nurses and physicians reported that the human resource services were not appropriate, which would hamper team members’ abilities to reach treatments goals (McGill and Felton, 2007). These factors, as well as nurses’ generally low scores on many team specific items and low degree of specialization may negatively affect the entire team and lead to disturbed diabetes management services. In the literature, the importance of nurses’ role as coordinators within teams, as well as their role in assessing patients’ diabetes control is emphasized (McGill and Felton, 2007). Therefore, clarity in the national guidelines with regard to the organization of the team for diabetes management and the roles of all team members would be well appreciated.

There was confusion among the staff regarding who the team leader was. This discrepancy may negatively impact the team dynamics and lead to disorganization and misconduct of the services provided (Barr and Dowding, 2015; Millward and Bryan, 2005).

The dietitians expressed concerns with the culture at primary care centers and the provision of the recommended diabetes management, as well as the effect of these challenges on patients’ decisions. Health educators had concerns with providing up-to-date knowledge and skills, their ability to respect patients’ beliefs, and being committed to providing the recommended diabetes management. This might not be well accepted by the patients and lead to poor compliance. Care providers should always consider patients’ beliefs in their team-based approach (Aschner *et al*., 2007b; 2010), as understanding the cultural influence on the disease is essential. Therefore, patients’ diabetic knowledge, skills, culture, and religious beliefs should be considered providers’ management tools; deviation from any of them may explain patients’ non-compliance with treatment plans (Lawrence and Rozmus, 2001; Hjelm *et al*., 2005; Abdulhadi *et al*., 2013). However, the complexity of modifying diabetic patients’ beliefs, norms, and behaviors in relation to promoting health, preventing ill health, and restoring health is largely depending also on cultural, social, and psychological factors. The evaluation of these values is not within the scope of this study.

Half of the physicians and many of the Omani team members said that nationality could affect patients’ decision related to diabetes management. This suggests that different nationalities of providers could act as a barrier for understanding and shared decision-making (González *et al*., 2010; Abdulhadi *et al*., 2013).

Senior and non-Omani professionals perceived themselves to have more team-related skills. This could be explained by their years of experience and training they received in their home countries. The same pattern was found among males compared with females, which may be in line with general perception of gender roles in Omani culture (Al-Lamky, 2007; Robbins *et al*., 2011).

According to all professionals, the infrastructure and work environment of the centers were helpful for team-based diabetes management. However, this contradicts the findings from physicians, nurses, dietitians, and health educators who perceived a shortage of human and technical resources. The laboratory resources and pharmacological treatments recommended in the national guidelines for diabetes management were available in all health centers; however, half of the centers could not provide cardiovascular drugs. Such deficiency may affect the patients’ diabetes management (Wens *et al*., 2005; Kheir *et al*., 2011). Among respondents, the majority of physicians indicated that they have easy access to the internet; though other groups noted limited access. If more computers were available at the centers, more up-to-date knowledge could be given to the patients. A few participants agreed that they were provided with financial support to update their diabetes knowledge. This may lead to job dissatisfaction and consequently, patient dissatisfaction with services. The answers that were received from physicians could be biased as the physicians’ group is of mixed nationalities and to declare that they do not have the necessary support may affect their presence in the centers (Firth-Cozens, 2004). It is also noteworthy that the physicians-in-charge and staff differed in their knowledge of the national diabetes guidelines, as the guidelines were not available to all staff. This contradicts the fact that the majority of the professionals revealed that they had up-to-date knowledge to conduct diabetes management clinics. This inconsistency may prevent the team-based diabetes management approach from being easily implemented.

### Strengths and limitations of the study:

This is a first study conducted in Oman and was designed to mirror the national guidelines for team-based diabetes management available at the time of data collection. The study looked into the perceptions of diabetes management clinic staff toward team-based approaches. It is a strength of the study that the data came from different sources, including registries and the survey which was collected from managers of all centers in Muscat as well as all care providers involved in diabetes management clinics.

This study involved a small number of health professions, particularly health educators and dieticians; therefore, the results should be interpreted with caution. Due to the small numbers, the results could not be given with high precision. Further studies, such as clinical audits, testing of health care providers’ knowledge and skills, or re-examination of providers’ competencies, are needed to confirm the accuracy of this study.

Further, this study included public primary health care centers in Muscat only; it did not include centers in other regions of Oman, or private sector settings. Therefore, the findings may not be applicable to other settings or regions of Oman. Though diabetes management clinics at the primary care centers are structured similarly throughout Oman, the study might be repeated to include all the public primary health care centers in all the regions of Oman.

In addition, the study focused only on the health service sector, while cultural and social aspects are also crucial for the success of diabetes management. Thus, studies focused on cultural, societal, and psychological perspectives of diabetes management and include patients, their families, and community perceptions are also needed.

## Conclusion

The study suggests important areas for potential improvement in public diabetes management clinics. The perceptions among team members towards diabetes management clinics at primary health care were diverse. All staff agreed to have high self-perceived competencies. Nurses showed low measures in self-perceived values and team-related skills, which are the basis for organization of teamwork. The centers are well equipped with laboratory support but not with pharmacological, technical, and human resources. These results are expected to be helpful in updating diabetes guidelines in Oman, which aim to improve the structure of diabetes teams and management of primary care workforces. This could include development of the guidelines regarding nurses’ availability and permanent assignment to their tasks in diabetes management, development of educational programmes for staff, and fair reallocation of diabetes management resources in the health sector. However, for a deeper understanding of cultural, social, and psychological factors that strongly relate to diabetes management, further studies on a larger scale and with other methodologies are also needed. In the long run this is expected to contribute to better health outcomes among the Omani diabetes population.

## Appendix

**Appendix 2:** The four themes (A: Self-perceived competencies, B: Self-perceived values, C: Self-perceived team-related skills and D: Self-perceived support/resources), the reported frequencies and percentages by the members of the diabetes management team for each of the 31 items of the survey in 26 centers in Muscat

		Physicians (*n*=91)			Nurses (*n*=80)			Dietitians (*n*=22)			Health Educators (*n*=19)			Total (*n*=212)		
	Themes	A (%)	D (%)	DNK NA	A (%)	D (%)	DNK NA	A (%)	D (%)	DNK NA	A (%)	D (%)	DNK NA	A (%)	D (%)	DNK NA
A	Self-perceived competencies															
1	I have up to date knowledge to conduct diabetes management clinic	84 (93)	5 (5.6)	1 (1.1)	72 (90)	6 (7.5)	2 (2.5)	19 (86)	3 (14)	0 (0)	18 (95)	1 (5.3)	0 (0)	193 (93)	11 (5.3)	3 (1.4)
2	I have up to date skills to conduct diabetes management clinic	86 (96)	3 (3.3)	1 (1.1)	74 (95)	4 (5.1)	0 (0)	19 (86)	3 (14)	0 (0)	19 (100)	0 (0)	0 (0)	198 (95)	10 (4.8)	1 (0.5)
4	I consider myself a resource person for the diabetic patient	80 (88)	5 (5.5)	6 (6.6)	68 (85)	12 (15)	0 (0)	20 (91)	2 (9.1)	0 (0)	17 (89)	2 (11)	0 (0)	185 (87)	21 (10)	6 (2.8)
B	Self-perceived values															
3	I am responsible to provide up to date knowledge and skills for diabetic patient	87 (96)	3 (3.3)	1 (1.1)	75 (94)	5 (6.3)	0 (0)	21 (95)	1 (4.5)	0 (0)	17 (89)	2 (11)	0 (0)	200 (94)	11 (5.2)	1 (0.5)
5	I perceive patient’s education as an important factor in diabetes management	91 (100)	0 (0)	0 (0)	77 (96)	3 (3.8)	0 (0)	22 (100)	0 (0)	0 (0)	19 (100)	0 (0)	0 (0)	209 (99)	3 (1.4)	0 (0)
6	I consider patient’s self-management an important part in diabetes management	88 (98)	1 (1.1)	1 (1.1)	75 (94)	5 (6.3)	0 (0)	22 (100)	0 (0)	0 (0)	19 (100)	0 (0)	0 (0)	204 (97)	6 (2.8)	1 (0.5)
7	I value communication with diabetic patients	91 (100)	0 (0)	0 (0)	79 (99)	1 (1.3)	0 (0)	22 (100)	0 (0)	0 (0)	19 (100)	0 (0)	0 (0)	211 (100)	1 (0.5)	0 (0)
8	I respect diabetic patient’s beliefs in regards to her/his disease	85 (94)	4 (4.5)	1 (1.1)	77 (96)	3 (3.8)	0 (0)	22 (100)	0 (0)	0 (0)	14 (74)	5 (26)	0 (0)	198 (94)	12 (5.7)	1 (0.5)
14	At my PHCC, we consider building PHCPs’ capacity very important to provide the recommended diabetes management	85 (93)	1 (1.1)	5 (5.5)	68 (87)	9 (12)	1 (1.3)	20 (91)	1 (4.5)	1 (4.5)	17 (89)	2 (11)	0 (0)	190 (90)	13 (6.2)	7 (3.3)
15	I am committed to MOH and PHCCs in terms of providing the recommended diabetes management	85 (94)	4 (4.4)	1 (1.1)	67 (86)	8 (10)	3 (3.8)	20 (91)	1 (4.5)	1 (4.5)	15 (79)	3 (16)	1 (5.3)	187 (89)	16 (7.7)	6 (2.9)
17	The culture of PHCCs in Oman helps me in providing the recommended diabetes management	66 (73)	23 (25)	2 (2.2)	61 (77)	15 (19)	3 (3.8)	12 (55)	10 (45)	0 (0)	14 (74)	5 (26)	0 (0)	153 (73)	53 (25)	5 (2.4)
27	As a professional provider, I have a good effect on patient’s decision related to diabetes management	84 (95)	3 (3.4)	1 (1.1)	72 (91)	5 (6.3)	2 (2.5)	20 (91)	1 (4.5)	1 (4.5)	19 (100)	0 (0)	0 (0)	195 (94)	9 (4.3)	4 (1.9)
28	My nationality can effect patient’s decision related to diabetes management	44 (50)	39 (44)	5 (5.7)	55 (70)	19 (24)	5 (6.3)	20 (91)	2 (9.1)	0 (0)	14 (74)	4 (22)	1 (5.3)	133 (64)	64 (31)	11 (5.3)
C	Self-perceived team-related skills															
18	At my PHCC, we provide team-based diabetes management	80 (88)	10 (11)	1 (1.1)	67 (85)	12 (15)	0 (0)	18 (90)	1 (5.0)	1 (5.0)	15 (79)	4 (21)	0 (0)	180 (86)	27 (13)	2 (1.0)
19	I consider myself part of a team when providing the recommended diabetes management	86 (95)	3 (3.3)	2 (2.2)	69 (88)	8 (10)	1 (1.3)	22 (100)	0 (0)	0 (0)	19 (100)	0 (0)	0 (0)	196 (93)	11 (5.2)	3 (1.4)
20	I consider myself a leader in the diabetes management team	66 (73)	20 (22)	5 (5.5)	50 (64)	27 (35)	1 (1.3)	9 (41)	13 (59)	0 (0)	11 (61)	7 (39)	0 (0)	136 (65)	67 (32)	6 (2.9)
21	I accept inter-professional relationship in the diabetes management team	88 (97)	0 (0)	3 (3.3)	73 (94)	5 (6.4)	0 (0)	20 (95)	1 (4.8)	0 (0)	18 (95)	1 (5.3)	0 (0)	199 (95)	7 (3.2)	3 (1.4)
22	I have a chance with my colleagues to reflect on patient’s decision related to diabetes management	82 (90)	8 (8.8)	1 (1.1)	63 (81)	14 (18)	1 (1.3)	20 (91)	1 (4.5)	1 (4.5)	17 (89)	2 (11)	0 (0)	182 (87)	25 (12)	3 (1.4)
23	I encourage teamwork related to diabetes management	90 (99)	0 (0)	1 (1.1)	75 (95)	4 (5.1)	0 (0)	22 (100)	0 (0)	0 (0)	18 (95)	1 (5.3)	0 (0)	205 (97)	5 (2.4)	1 (0.5)
24	I have the ability to deliver teamwork related to diabetes management	87 (96)	3 (3.3)	1 (1.1)	71 (90)	8 (10)	0 (0)	21 (95)	0 (0)	1 (4.5)	18 (95)	1 (5.3)	0 (0)	197 (93)	12 (5.7)	2 (0.9)
25	I value teamwork related to diabetes management	87 (96)	3 (3.3)	1 (1.1)	71 (90)	6 (7.6)	2 (2.5)	21 (95)	0 (0)	1 (4.5)	18 (95)	1 (5.3)	0 (0)	197 (93)	10 (5.7)	4 (1.9)
26	I encourage collaboration action in related to diabetes management	86 (98)	0 (0)	2 (2.3)	71 (90)	6 (7.6)	2 (2.5)	21 (100)	0 (0)	0 (0)	19 (100)	0 (0)	0 (0)	197 (95)	6 (2.9)	4 (1.9)
D	Self-perceived support/resources															
9	I ensure that I have time to update my diabetes knowledge and skills	83 (91)	6 (6.6)	2 (2.2)	63 (79)	17 (27)	0 (0)	19 (86)	3 (14)	0 (0)	16 (84)	3 (16)	0 (0)	181 (85)	29 (14)	2 (0.9)
10	I have an easy access to the INTERNET to update my diabetes knowledge	76 (84)	12 (13)	3 (3.3)	52 (66)	26 (33)	1 (1.3)	9 (41)	12 (55)	1 (4.5)	12 (63)	7 (37)	0 (0)	149 (71)	57 (27)	5 (2.4)
11	I have been supported financially to update my diabetes knowledge (attending courses in diabetes)	39 (43)	44 (48)	8 (8.8)	27 (35)	45 (58)	6 (7.7)	7 (33)	13 (62)	1 (4.8)	7 (37)	12 (63)	0 (0)	80 (38)	114 (55)	15 (7.2)
12	My manager (MOIC) supports my diabetes knowledge update	84 (92)	5 (5.5)	2 (2.2)	71 (90)	8 (10)	0 (0)	19 (86)	1 (4.5)	2 (9.1)	17 (89)	2 (11)	0 (0)	191 (91)	16 (7.6)	4 (1.9)
13	My colleagues support my diabetes knowledge update	81 (89)	4 (4.4)	6 (6.6)	71 (90)	7 (8.9)	1 (1.3)	20 (91)	0 (0)	2 (9.1)	17 (89)	2 (11)	0 (0)	189 (90)	13 (6.2)	9 (4.3)
16	According to me, the infrastructure of PHCCs in Oman helps in providing the recommended diabetes management	65 (71)	26 (29)	0 (0)	63 (80)	13 (16)	3 (3.8)	10 (45)	12 (55)	0 (0)	14 (74)	5 (26)	0 (0)	152 (72)	56 (27)	3 (1.4)
29	At my PHCC, work environment is helpful in delivering the recommended diabetes management	73 (83)	14 (16)	1 (1.1)	64 (81)	14 (18)	1 (1.3)	16 (73)	6 (27)	0 (0)	12 (63)	7 (37)	0 (0)	165 (79)	41 (20)	2 (1.0)
30	At my PHCC, we have the appropriate human resources to conduct the recommended diabetes management clinic	59 (67)	28 (32)	1 (1.1)	54 (68)	25 (32)	0 (0)	17 (77)	3 (14)	2 (9.1)	15 (79)	3 (16)	1 (5.3)	145 (70)	59 (28)	4 (1.9)
31	At my PHCC, we have the appropriate technical resources to conduct the recommended diabetes management clinic	55 (63)	32 (36)	1 (1.1)	42 (53)	34 (43)	3 (3.8)	15 (68)	6 (27)	1 (4.5)	13 (68)	5 (26)	1 (5.3)	125 (60)	77 (37)	6 (2.9)

Denominator of each item is equal to the total number of respondents to that item. PHCC/s=primary health care center/s; A=agree=(agree+completely agree); D=disagree=(disagree+completely disagree); PHCP/s=primary health care provider/s; DNK=don’t know; MOIC=medical officer in charge; NA=not applicable; MOH=Ministry of Health.
